# Attendance, activation and health profiles of participants, a prospective study on a regional cardiometabolic disease self-management program in Laval, Canada

**DOI:** 10.1186/s12889-021-10558-6

**Published:** 2021-03-12

**Authors:** Magali Girard, Janusz Kaczorowski, Marie-Thérèse Lussier, Vivianne Martin

**Affiliations:** 1 University of Montreal Hospital Research Centre (CRCHUM), 850, rue St-Denis, Montreal, Canada; 2 Department of Family and Emergency Medicine, University of Montreal, Montreal, Canada; 3 CIUSSS du Centre-Sud-de-l’Île-de-Montréal, Montreal, Canada

**Keywords:** Chronic disease, Self-management programs, Health promotion, Volunteers, Health profile, Patient activation measure

## Abstract

**Background:**

Chronic diseases are responsible for over 70% of all deaths globally. While some self-management programs have been shown to be efficacious in preventing or altering trajectories for some chronic conditions, scaling-up and sustaining such programs beyond tightly-controlled study conditions remain a major challenge. CISSS-Laval partnered with the Cardiovascular Health Awareness Program team to co-develop Cible-santé/prévention and evaluate the first cohort of participants enrolled in the program, in order to better understand the program’s implementation and scope. The objective of the current study was to describe the profile of attendees and the level of engagement of participants in a new, region-wide cardiometabolic disease self-management program offered in Laval, Canada.

**Methods:**

This was a prospective study with no comparison group. Potential participants were identified and referred to the program from April to December 2015 by their primary care health professional practicing in one of the city’s interdisciplinary primary care clinics. They had their blood pressure, waist circumference and body mass index measured by trained volunteers, and completed a questionnaire on health habits, level of activation and the risk of developing prediabetes and type 2 diabetes over the next 10 years.

**Results:**

A descriptive analysis of the first cohort of 141 Cible-Santé/prévention participants showed very low attendance. Furthermore, only 1 in 10 of enrolled participants completed the full program. The program typically attracted adults with some risk factors associated with their conditions (high waist circumference, obesity), but with an already high level of knowledge, skills and confidence to participate in self-management activities.

**Conclusion:**

This study provides a portrait of new participants to a self-management cardiometabolic disease program, which highlights the potential of supporting patients ready to make changes but also exposes the difficulty of attracting a larger number and diversity of participants and in encouraging completion of the program.

**Supplementary Information:**

The online version contains supplementary material available at 10.1186/s12889-021-10558-6.

## Background

Chronic diseases — including cardiovascular diseases, cancers, chronic respiratory diseases and diabetes — are responsible for over 70% of all deaths globally. Modifiable risk factors that include smoking, physical inactivity and unhealthy diets are the main drivers behind the growing epidemic of chronic diseases [1]. While some health promotion and self-management programs have been shown to be efficacious in preventing or altering trajectories for some chronic conditions, in modifying risk profiles and in improving satisfaction and knowledge of participants [2], scaling-up and sustaining such programs beyond tightly-controlled study conditions remain a major challenge.

Participation rates in health promotion and chronic disease self-management programs are generally low [3], despite their wide availability in most high-income countries, often not requiring referrals from a health professional and offered free of charge or at low cost [4]. People who engage in health promotion or self-management programs are more likely to be female [5], younger [4, 6, 7], more educated [6, 8], of higher socioeconomic status [9], Caucasian [5], in better physical health [3, 6, 7] and to already be adhering to healthier lifestyles [10]. They are also more likely to have a better understanding of their condition than patients who do not participate [6].

Program type and program location also influence attendance. The participation rates are higher when programs are offered in a variety of time and place settings, when there is no need for referral and when participation is free or low cost [5]. Elzen and colleagues showed that living far away from a program’s location and transportation constraints both represented barriers to participation [10]. Another study found that living in rural areas with limited access to physicians increased utilization of a diabetes self-management education program [4].

The objective of the current study was to describe the profile of attendees and the level of engagement of participants in a new, region-wide cardiometabolic disease self-management program offered in Laval, Canada. The study took place in 2015 and looked at the first cohort of Cible-Santé/prévention participants.

## Method

### Design

This was a prospective study with no comparison group.

### Date and setting

The intervention took place from April to December 2015 in one of the Local community services centres of Laval, a large predominantly French-speaking suburban community in the Greater Montreal region of the province of Quebec, Canada.

### Participants

To be eligible for Cible-Santé/prévention program, patients had to be registered with one of Laval’s interdisciplinary primary care clinics (*Groupe de médecine de famille* or GMF), had to be 18 years old or over, understand French and had to have at least one of the following conditions: type 2 diabetes, pre-diabetes, hypertension, pre-hypertension, cardiovascular disease, dyslipidemia, stroke/transient ischemic attack, heart failure, kidney failure, metabolic syndrome or peripheral vascular disease. There were no exclusion criteria.

### Intervention

The Cible-Santé/prévention program was developed by the regional health authority, Centre intégré de santé et des services sociaux de Laval (CISSS-Laval) servicing over 400,000 residents of Laval, the second largest city in the province of Québec, Canada. The goal of the program was to help participants modify some of their unhealthy lifestyles in order to support the self-management of their cardiometabolic diseases. The program was led by an interdisciplinary team (nutritionists, kinesiologists and nurses) trained in motivational interviewing techniques [11, 12]. The SMART objectives developed by participants were at the heart of the change process. The 8-month program included three group workshops (one per month: nutrition, physical activity, and tobacco and stress management) and five individual follow-ups (provided by health professional and according to the set objective).

CISSS-Laval partnered with the Cardiovascular Health Awareness Program team to co-develop Cible-santé/prévention and evaluate the first cohort of participants enrolled in the program, in order to better understand the program’s implementation and scope. CHAP is a patient-centred, interdisciplinary, multi-faceted, community-led, cardiovascular disease prevention and management program targeting older Canadian adults. It was felt that a more detailed health profile of new participants could assist CISSS-Laval managers to optimize the recruitment strategies to make the program available to a wider range of participants and to better meet their needs. These findings might also offer useful insights that can inform similar future programs.

Potential participants were identified and referred to the program by their primary care health professional practicing in one of the city’s interdisciplinary primary care clinics. Referral to the program was at the discretion of the health care professional, i.e., they were not required to refer all eligible patients or even a minimum number of patients. Referrals were sent to CISSS-Laval appointment centre, who subsequently contacted potential participants to enrol them into the program.

Cible-Santé/prévention participants were invited to attend three group-based workshops and five individual follow-up sessions with a nutritionist, kinesiologist or nurse over an eight-month period. At their first group workshop, all first Cible-Santé/prévention cohort patients were invited to participate in a CHAP evaluation session.

Building on 20 years of work around CHAP [13–22], the evaluation consisted of physical measurements that included blood pressure, height, weight and waist circumference, a questionnaire on healthy habits, level of activation and assessment of prediabetes and type 2 diabetes risk at the onset of the first Cible-Santé/prévention group session. The CHAP sessions were run by locally recruited and trained volunteers and supervised by public-health nurses.

### Variables

Data on demographic characteristics, health status, risk factors and patient activation were collected at baseline and were self-reported by participants (See questionnaire in Additional file 1).

The classification of age groups is based on that of the CANRISK questionnaire, with the difference that the first category includes all respondents under 45 years of age and the upper category includes all respondents over 65 years of age. The CANRISK Questionnaire was developed and tested for a population aged 45–74, while our study included participants 18 years of age and older.

Blood pressure categories were derived from the Hypertension Canada’s guideline for blood pressure measurement, diagnosis, assessment of risk, prevention and treatment of hypertension [23]. A high blood pressure is defined as systolic blood pressure over 140 mmHG.

For physical activity, patients were asked: “In a typical week, what kind of physical activity do you do? Write down all the physical activities in which you are out of breath, even slightly, and indicate how many minutes you do, including leisure, transportation or work-related activities. Examples of physical activities: Jogging, aquafitness, social dancing, walking, biking, running, skiing, swimming, gardening, or working outdoors, etc.” (Additional File 1). Respondents had to fill up a table to provide the type of activity and number of minutes of physical activity for each day of a typical week. The metabolic equivalents (METs) were calculated using the Compendium of Physical Activity [24], which classifies a multitude of physical activities (from gardening to swimming) according to an intensity scale: MET values. The more intense is an activity, the higher the MET is. In the results section, we present the equivalence in minutes of slow walking per day to facilitate the interpretation and presentation of results.

The validated Patient Activation Measure (PAM) is a 13-item survey that assesses the knowledge, skills and confidence for self-management of one’s own health, and was developed by Dr. Judith Hibbard, Dr. Bill Mahoney and colleagues at the University of Oregon [25]. To calculate the score, we followed the guidelines given by the license provider. Four levels of activation were categorized according to the score ranging from 0 to 100. At Level 1, the person is considered “unaware”, does not feel confident enough and is a passive recipient of care. At Level 2, the person lacks confidence and understanding of their health care (e.g., how to take or know what their medications do). At Level 3, the person knows the essential facts and begins to take steps to take care of their health, but may lack the confidence and skills to take complete control of their health care. At Level 4, knowledge, skills and confidence for self-management are all present.

All respondents who reported not being diagnosed with type 2 diabetes or being unsure were asked to complete the Canadian Diabetes Risk Questionnaire (CANRISK). Developed by the Public Health Agency of Canada, this validated questionnaire-based tool assesses the risk of developing pre-diabetes and type 2 diabetes in the next 10 years [26]. Each answer to this 12-question survey has a point value (e.g. 0 point if between 40 and 44 years old; 15 points if between 65 and 74 years old). The total number of points represents the total score of a respondent. Respondents with a score lower than 21 are at low risk of developing prediabetes or type 2 diabetes over the next 10 years; 21 to 32 points are at a moderate risk, and between 33 to 42 are at high risk, and respondents with a total score higher than 42 are at very high risk.

During the sessions, physical measurements were taken by trained volunteers and included waist circumference, weight and height (to calculate the body mass index), as well as participant blood pressure, using an automated measuring device, the Microlife WatchBP Office Afib, which allows dual-cuff measurement and atrial fibrillation detection (Additional File 1). The participants with elevated blood pressure (> 140/90 mmHg) were assessed by the nurse and referred to their family physician for follow-up.

Program participation was defined as completing all three group-based workshops and five individual follow-ups. Program completion rate was calculated using the total number of participants who registered as the denominator.

### Statistical methods

Data were analyzed with SPSS, version 24.0. Univariate descriptive statistics and frequency distributions were used to summarize the data of individuals who participated in a CHAP session. The study protocol was approved by the scientific and research ethics committee of the Laval regional health authority (CISSS: Centre intégré de santé et de services sociaux de Laval).

## Results

**Fig. 1:**
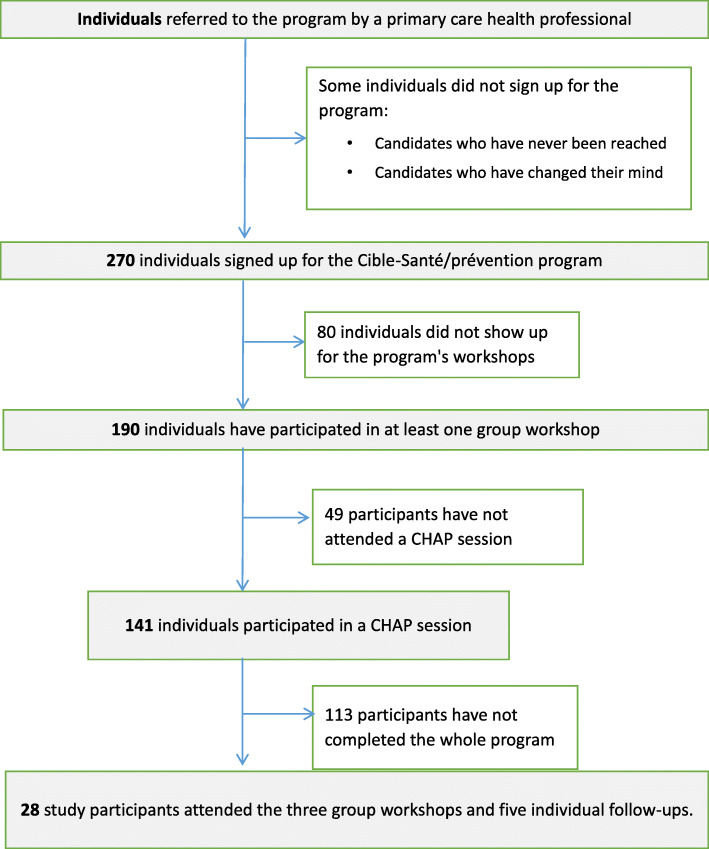
Flow chart of participant recruitment. Shows the number of participants at each stage of recruitment

The total number of patients referred by health professionals practicing in one of the GMFs in Laval has not been recorded. Participants were initially referred by their primary care health professional (this number was not recorded). Afterwards, 270 participants registered for Cible-Santé/prevention (Fig. 1). Of these, 190 attended at least one group-based workshop and 141 took part in the CHAP evaluation session that preceded the first group workshop. One in ten (10.4%, 28/270) of the enrolled participants had attended all three group workshops and five individual follow-ups.

Tables 1 to 4 present data on individuals who participated in a CHAP session (*n* = 141). More than half of the participants were 55 years of age or older, and the majority were female (Table 1).

**Table 1:** Characteristics of individuals who participated in a CHAP session

	n	%
**Age** (*n* = 140, average 59 years-old)		
18 to 45 years-old	15	10.7
45 to 54 years-old	34	24.3
55 to 64 years-old	41	29.3
65 years-old and over	50	35.7
**Gender** (*n* = 141)		
Male	55	39.0
Female	86	61.0
**Waist Circumference** (*n* = 136)		
F < 80 cm | H < 94 cm (Low Risk)	7	5.1
F80-88 cm | H < 94-102 cm (High Risk)	16	11.8
F > 88 cm | H > 102 cm (Very High Risk)	113	83.1
**BMI** (n = 140)		
Normal (< 25)	9	6.4
Overweight (25–29)	34	24.3
Obese (30–34)	57	40.7
Severely Obese (35–39)	25	17.9
Morbid Obese (40 et plus)	15	10.7
**Systolic Blood Pressure** (n = 140, average = 136.51)		
Less than 120	18	12.9
120 to 139	73	52.1
140 to 159	39	27.9
160 and over	10	7.1
**Diastolic Blood Pressure** (n = 140, average = 78.93)		
Less than 80	78	55.7
80 to 84	30	21.4
85 to 89	15	10.7
90 and over	17	12.1

**Table 2:** Lifestyle Habits

	n	%
**Daily consumption of fruit and vegetables** (*n* = 138, mean (SD) = 5)		
1 to 3 portions	41	29.7
4 to 6 portions	69	50.0
7 to 9 portions	24	17.4
10 portions and over	4	2.9
**Physical Activity** (*n* = 131)		
Inactive	24	18.3
Very light (< 20 min walk /day)	22	16.8
Light (20 to 40 min walk /day)	42	32.1
Moderate (40 to 60 min walk /day)	27	20.6
Vigorous (> 1 h walk /day)	16	12.2
**Smoking Status** (n = 141)		
Smoker	13	9.2
None smoker	128	90.8
**Stress day per week** (*n* = 126)		
Rarely or never	60	47.6
Sometimes (1–2 day/week)	18	14.3
Often (3–4-5 day/week)	34	27.0
Regularly (6–7 day/week)	14	11.1

**Table 3:** Patient Activation Measure (n = 141, average = 65.65)

	n	%
Level 1: Disengaged and overwhelmed	9	6.4
Level 2: Becoming aware but still struggling	20	14.2
Level 3: Taking action and gaining control	43	30.5
Level 4: Maintaining behaviours and pushing further	69	48.9

**Table 4:** Diabetes and CANRISK Score

	n	%
**Self-reported diabetes diagnosis** (n = 141)		
Diabetic	68	48.2
Non-diabetic	64	45.4
Unsure	9	6.4
**Score CANRISK** (*n* = 72/73 did not declare being diagnosed with diabetes or unsure)		
Low Risk (score < 21)	3	4.2
Average Risk (21 to 32)	24	33.3
High Risk (33 to 42)	34	47.2
Very High Risk (> 42)	11	15.3

The vast majority of participants (83.1%, 113/136) had a waist circumference of more than 88 cm for women and more than 102 cm for men, and nearly 70% (97/140) were classified as obese. Over 17% had a diagnosis of hypertension (not shown in table) and more than a third had blood pressure above > 140/ 90 mmHg).

Approximately half of participants reported eating the recommended daily servings of fruits and vegetables (four to six servings), and over a third of participants reported very little to no physical activity (16.8 and 18.3%) (Table 2). Nearly half of the participants were classified at a level 4 of activation (Table 3).

Of all 141 participants who had participated in a CHAP evaluation session, 68 (48.2%) reported being diabetic (Table 4). Those who had no diagnosis or who were unsure (*n* = 73) completed the CANRISK. More than 60% were classified as having high or very high risk of developing pre-diabetes or diabetes.

## Discussion

The evaluation of the first cohort of Cible-Santé/prévention participants showed very low attendance (270 adults who registered from over 200,000 patients^1^ registered in all of Laval’s GMFs). Furthermore, only 1 in 10 of enrolled participants completed the full program. While the total number of patients referred to the program was not recorded, given the prevalence of targeted conditions and risk factors in primary care, the number of patients who registered and completed the program was quite low. These results are consistent with what has been reported in the literature [3]. The literature also suggests that participation is higher when the program is offered for free [5] and not far away from home [10]. Although the program was offered free of charge by the local health authorities and in settings familiar to participants, we propose several explanations for the low attendance and completion rates.

First, Cible-Santé/prévention was a new program introduced in the already large spectrum of services provided by the regional health authority. Given that this was the first cohort of participants, a more comprehensive communication strategy might help raise awareness about the program and its goals. Second, the evidence suggests that when programs are offered in a variety of time and place settings, participation rates are typically higher [5]. Cible-Santé/prévention was, at the time, offered to one new cohort per month and this study focused only on the first cohort. Third, the low completion rate could be explained by the long duration of the program (8 months) and low frequency of interactions with health professionals (one group workshop or individual follow-up per month), which may have de-motivated some participants. A telephone follow-up was conducted with Cible-Santé/prévention participants who had not completed the program [27]. Most respondents (*n* = 41) abandoned the program due to lack of motivation; some felt they had achieved their goals already and were satisfied with the changes made.

The profile of Cible-Santé/prévention participants suggests that they tend to engage in a relatively healthy lifestyle. This is also consistent with what is found in the literature, which suggests that people who engage in health promotion or self-management programs are more likely to already be adhering to healthier lifestyles [10]. It is difficult to establish to what extent these self-reported “healthy habits” represent a true reflection of reality in contrast to a desire to provide socially acceptable answers. However, the CHAP sessions contributed objective physical measures data as well as validated questionnaire data. These results tend to confirm the appropriateness of the healthcare provider referrals to this cardiometabolic disease self-management program.

Nearly half of participants were considered highly activated (Level 4 of the PAM). People who engage in these types of programs are more likely to have a better understanding of their condition than patients who do not participate [6]. Since health professionals were not required to refer all eligible patients and could choose who to refer, a “selection bias” is possible since physicians may have referred more patients they perceived as “ready” to engage in this type of program. Given that after referral from a health professional people could self-select to participate in the Cible-Santé/prévention program, high levels of activation are not surprising. Their participation in the program is probably the result of their high level of knowledge, skills and confidence.

A limitation of the study is the lack of data regarding the number of professionals who referred their patients to the program, and the reasons for never attending a Cible-Santé/prevention workshop after registering. This study was conducted in a real care setting and in collaboration with program managers, which explains the absence of certain data (missing denominator). This would have allowed us to know the proportion of referred patients who subsequently enrolled in the program, and thus know the number of patients who follow-up on such referrals.

Another limitation of this study is the lack of a control group. Having a comparison group with similar baseline characteristics, that would not have participated in this chronic disease self-management program or maybe enrolled in a different program, would have enabled us to better assess the characteristics associated with recruitment and participation.

Finally, some measures were self-reported by respondents, including physical activity level, fruit and vegetable consumption, and smoking status, which may have influenced the validity of the responses given. Otherwise, trained volunteers under the supervision of a nurse took physical measurements, including blood pressure, body mass index and waist circumference, on-site.

The study was the first evaluation of this new regional cardiometabolic disease self-management program. It was intended to describe attendance, activation and health profiles of first-cohort participants, and can serve as a precursor to further testing of the program. An evaluation of its effectiveness and impact on health outcomes and behaviours would help determine whether the Cible-Santé/prévention needs to be continued in its actual form, be redesigned or abandoned.

## Conclusion

The evaluation by the CHAP team made it possible to draw a health profile of the first cohort of Cible-Santé/prévention participants. CHAP volunteers were trained to adequately measure blood pressure, body mass index and waist circumference, and to assist participants in completing their questionnaires. The results showed a very low attendance and program completion rate. The program typically attracted adults with some risk factors associated with their chronic conditions (high waist circumference, obesity), but with an already high level of knowledge, skills and confidence to participate in self-management activities.

This study provides a portrait of new participants to a cardiometabolic disease self-management program, which highlights the potential of supporting patients ready to make changes but also exposes the difficulty of attracting a larger number and diversity of participants and in encouraging completion of the program. Further studies are needed to assess the effect of the program on self-management skills and health outcomes of participants, test several recruitment strategies to increase the diversity of the target population and to compare participants’ health profiles with non-participants.

## Supplementary Information

**Additional file 1.**

## Data Availability

The datasets used and/or analysed during the current study are available from the corresponding author (MG) on reasonable request.

## Abbreviations

BMI: Body Mass Index
CANRISK: Canadian Diabetes Risk Questionnaire
CHAP: Cardiovascular Health Awareness Program
CISSS-Laval: Centre intégré de santé et services sociaux de Laval
GMF: *Groupe de médecine de famille* (Interdisciplinary primary care clinics)
METS: Metabolic Equivalents
PAM: Patient Activation Measure
SMART: Specific, Measurable, Attainable, Relevant, and Time-Bound

## Footnotes

1 This is an estimation of the number of patients registered in one of Laval’s GMFs at the time of data collection. We don’t know the total number of patients who met the eligibility criteria of Cible-Santé/prévention.
