# Supplementary material for: Attendance, activation and health profiles of participants, a prospective study on a regional cardiometabolic disease self-management program in Laval, Canada
Source: BMC Public Health. 2021 Mar 12;21:497. doi: 10.1186/s12889-021-10558-6 (PMC7953555; doi:10.1186/s12889-021-10558-6)
Supplement: Supplementary file 1 — Additional file 1. [file 12889_2021_10558_MOESM1_ESM.docx]

| \| **Name of participant** : ____________________________________ \| Date :  ____ / ____ / ____ (dd/mm/yyyy) \| \| --- \| --- \|   **Measurement of blood pressure, body mass index and waist circumference** | | |
| --- | --- | --- | --- | --- |
| 1. Blood pressure and pulse measurement (average of 3 measurements) : | | |
| LEFT arm :  Systolic ________  Diastolic _______ | RIGHT arm :  Systolic ________  Diastolic _______ | Pulse (b/m) : __________________  Cuff size :   - Medium - Large - Extra large |
| 1. Risk of atrial fibrillation: | | - Yes - No - Not applicable |
| 1. Body Mass Index (BMI) : ______________ | | Weight : ____________   - Kg - Lbs   Height : ____________   - cm - inches |
| 1. Waist circumference : _________________ | | - cm - inches |

**Assessment of current lifestyle habits**

We would like to know your current lifestyle habits. This questionnaire will allow us to get to know you better. Please answer to the best of your knowledge.

**Diet**

1. In general, how many servings of fruits AND vegetables do you eat each day?

| To help you assess your fruit and vegetable intake, here are some examples of a serving size:   - a fruit or vegetable the size of a tennis ball - 250 ml (1 cup) lettuce - 125 ml (½ cup) of fruit or 100% fruit juice - 125 ml (½ cup) of vegetables or vegetable juice - 60 ml (¼ cup) of dried fruit   Note: Fruits and vegetables in a mixed dish also count (e.g. vegetable soup, stuffed peppers). |
| --- |

| Meals or snacks | Number of portions of fruit and vegetables |
| --- | --- |
| Breakfast |  |
| Lunch |  |
| Diner |  |
| Snacks (morning, afternoon and evening) |  |

**Physical Activities**

1. In a typical week, what kind of physical activity do you do?

| Write down all physical activities during which you are out of breath, even slightly, and specify how many minutes you do, including leisure, transportation or work-related activities.  Examples of physical activities: Jogging, aquafitness, social dancing, walking, biking, running, skiing, swimming, gardening or outdoor work, etc. |
| --- |

|  | Type of physical activity | Number of minutes during which the activity is performed |
| --- | --- | --- |
| Monday |  |  |
| Tuesday |  |  |
| Wednesday |  |  |
| Thursday |  |  |
| Friday |  |  |
| Saturday |  |  |
| Sunday |  |  |

**Tobacco**

1. Do you smoke cigarettes, electronic cigarettes, cigars, cigarillos, pipes or chicha?

- Yes
- No

1. If yes, how often and how much do you usually smoke?

|  | Number of cigarettes | E-cigarettes* | Number of cigars | Number of cigarillos | Number of pipes | Number of chicha |
| --- | --- | --- | --- | --- | --- | --- |
| Everyday |  |  |  |  |  |  |
| Occasionally |  |  |  |  |  |  |

* If e-cigarette, simply specify whether you smoke every day or occasionally.

**Stress**

1. On average, how many days a week do you feel stressed or overwhelmed because of your responsibilities?

Number of days: _______

- No, not in the last 6 months
- No, rarely or never
